# Metabolic Regulation of Innate Lymphoid Cell-Mediated Tissue Protection—Linking the Nutritional State to Barrier Immunity

**DOI:** 10.3389/fimmu.2017.01742

**Published:** 2017-12-07

**Authors:** Christoph Wilhelm, Schekufe Kharabi Masouleh, Alexander Kazakov

**Affiliations:** ^1^Unit for Immunopathology, Department of Clinical Chemistry and Clinical Pharmacology, University Hospital Bonn, University of Bonn, Bonn, Germany

**Keywords:** innate lymphoid cells, metabolic syndrome, allergic inflammation, immunometabolism, aryl hydrocarbon receptor, vitamin A, lipid mediators

## Abstract

Innate lymphoid cells (ILC) are a recently described group of tissue-resident immune cells that play essential roles in maintaining and protecting the tissue barrier against invading pathogens. Extensive research has revealed that ILC-mediated immune responses are controlled by dietary components and metabolites. An additional role of ILC as important direct regulators of host metabolism and glucose tolerance is emerging. This suggests that ILC may act as key dietary sensors integrating nutritional and metabolic stress to facilitate both maintenance of barrier sites and a coordinated immune response protecting these tissues. In this respect, investigations have begun to determine how different ILC responses are metabolically fueled and the impact of nutrient availability on the regulation of ILC function. Here, we discuss the current literature concerning dietary and metabolic control of ILC. In particular, we address whether the dietary and metabolic control of ILC and their simultaneous influence on host metabolism may function as a coordinated program of barrier defense.

## Introduction

Innate lymphoid cells (ILC) have recently emerged as an integral component of tissue immunity with several subsets described both in mice and man (1, 2). Type 1 ILC (ILC1) including NK cells express the transcription factor T-bet, produce the cytokine interferon (IFN)-γ, and are implicated in protection against intracellular pathogens such as *Toxoplasma gondii* (3, 4). ILC2 express the transcription factor GATA-3 and produce the cytokines interleukin (IL)-5, IL-9, IL-13, and amphiregulin, promoting immunity against helminth infections and tissue repair but also allergic inflammation and asthma (1, 5). By contrast, ILC3 are characterized by the expression of the transcription factor RORγt and the cytokines IL-22 and IL-17 (1). Both cytokines mediate anti-bacterial immune responses and prevent bacterial translocation across barriers. However, aberrant regulation of ILC3 and in particular the expression of IL-17 is a potential driver of chronic gastrointestinal inflammation (1, 6, 7). Exposure to cytokines results in the activation of ILC and both IL-12 and IL-18 stimulate ILC1, the epithelial cell-derived cytokines IL-25, IL-33, or TSLP lead to the activation of ILC2, while ILC3 readily respond to IL-1 and IL-23 with the production of IL-22 (1).

Current data suggest that ILC may play an additional role by maintaining metabolic regulation and glucose tolerance of the host (8–10). Simultaneously, specific ILC functions appear to be coupled to the availability of nutrients and diet-derived factors (11–18). Thus, the function of ILC may extend beyond the simple maintenance of barrier surfaces to the maintenance of body homeostasis and metabolic control of the organism. Here, we discuss the function of ILC in the context of these new findings.

## Control of ILC Barrier Function by Dietary-Derived Products

The intestinal immune system is equipped to directly sense and react to dietary nutrients and substances (19). This sensing is largely dependent on the expression of specialized receptors many of which belong. This includes receptors for the recognition of vitamin A and tryptophan metabolites, the retinoic acid receptors (RAR) and aryl hydrocarbon receptor (AhR), respectively. Of note, both receptors are expressed in ILC suggesting a particular importance of these pathways for ILC-mediated barrier immunity.

### Vitamin A

**Figure 1:**
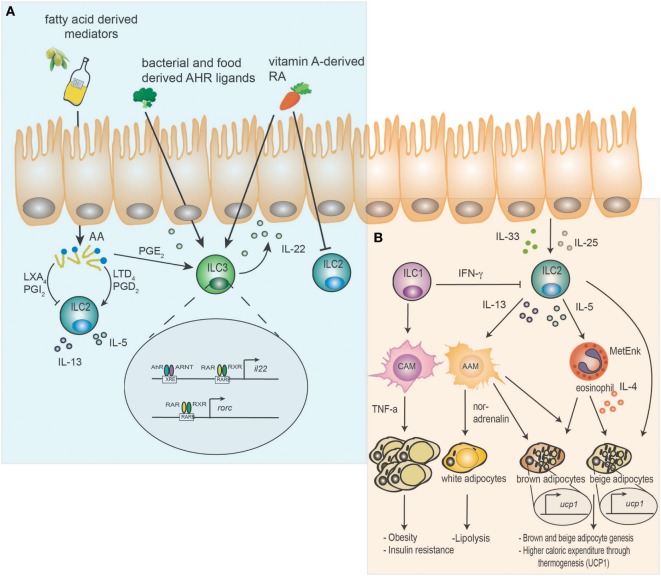
Dual function of innate lymphoid cells (ILC) as relays of dietary components and host metabolism. **(A)** Dietary components such as aryl hydrocarbon receptor (AhR) ligands and vitamin A-derived retinoic acid (RA) are sensed by ILC. AhR/ARNT signaling promotes interleukin (IL)-22 release from ILC3 by directly binding to xenobiotic response elements in the *il22* locus. Vitamin A-derived RA additionally enhances IL-22 secretion by binding of retinoic acid receptor (RAR) and retinoic X receptor (RXR) to retinoic acid response elements on the DNA and induces rorc expression. Furthermore, retinoic acid (RA) can directly inhibit ILC2. Essential plant-derived fatty acids (FA) are converted into arachidonic acid (AA) in the host and further metabolized into prostaglandins (PGs), leukotrienes (LTs), and lipoxins (LXs). Prostaglandin D_2_ (PGD_2_) and leukotriene D_4_ (LTD_4_) activate ILC2 and support expression of the cytokines IL-5 and IL-13. In contrast prostaglandin I_2_ (PGI_2_) and lipoxin A_4_ (LXA_4_) inhibit the function of ILC2. Prostaglandin E_2_ (PGE_2_) supports the function of ILC3 and IL-22 expression. **(B)** Epithelial-derived cytokines IL-33 and IL-25 activate ILC2 to release methionine-enkephalin (MetEnk), IL-13 and IL-5, which promote the activation of alternatively activated macrophages (AAM) and eosinophils. IL-4 secreted by eosinophils and AAM enhance brown and beige adipocyte genesis and mediate higher caloric expenditure through induction of uncoupling protein 1 (UCP1). Additionally, AAM may promote lipolysis in white adipocytes *via* noradrenalin. ILC2 are inhibited by ILC1 derived interferon (IFN)-γ, which drives classically activated macrophages (CAM) and the development of obesity and insulin resistance in a TNF-α-dependent manner.

Vitamin A is a fat-soluble essential micronutrient obtained through the diet as carotenoids (in fruits and vegetables), or as vitamin A itself in the form of retinyl esters (in foods of animal origin) (20). RA is required for the growth and development of the organism but also has profound effects on the immune system controlling innate and adaptive immune responses (19, 21–23). Lately, the essential effects of RA in control of ILC3 responses were revealed (14–16, 24). Animals deficient in vitamin A, display reduced numbers of ILC3 in contrast to mice fed vitamin A. This reduction in ILC3 has functional consequences for intestinal immunity, as these mice are more susceptible to infection with the bacterial pathogen *Citrobacter rodentium* than vitamin A competent animals (15). This was primarily due to a lack of ILC3-mediated IL-22. Conversely, delivery of exogenous RA drives production of IL-22 from ILC3, which in turn protects against *C. rodentium* and ameliorates the pathology of DSS-induced colitis (14). RA appears to control ILC3 on multiple levels. Activated RAR binds directly to *il22* and *rorc* promoter regions in ILC3, as well as promoting transcriptional activity of genes important for ILC3 development and function (14, 16). Moreover, homing of ILC3, but not ILC2, to the gut depends on RA, which upregulates CCR9 and α_4_β_7_ (25). Finally, this axis appears to be active in humans, since ILC3 differentiation, IL-22 production, and RORγt-mediated conversion of human ILC1 to ILC3 also relies on RA (26). The effects of vitamin A deficiency extends to development of the immune system, as inhibition of maternal uptake of retinoids early in ontogeny impairs the development of lymphoid tissue inducer cells and, thus, the development of secondary lymphoid organs of the offspring (16). In addition to its effects on ILC3, RA suppresses ILC2 through downregulation of IL-7Rα (15). Although, the molecular basis and why this mechanism is only affecting ILC2 is unknown, increased IL-7Rα expression in the absence of RA may enable ILC2 to compete more efficiently for limiting intestinal IL-7. This may be particular effective since the maintenance and differentiation of ILC3 is simultaneously decreased. Thus, vitamin A deficiency induces an adaptation of the intestinal immune response by adjusting the proportions of ILC2 and ILC3, resulting in expansion of IL-13 producing ILC2 and efficient expulsion of helminth (15) (Figure 1A). This enables maintenance of the intestinal barrier in the context of chronic parasite infections, but not bacterial infections.

### Ligands Activating the AhR

Ahr, best known for its ability to metabolize environmental toxins, was recently shown to directly effect immune cells, including ILC3 through tryptophan-derived metabolites (11–13, 27). These metabolites are formed either exogenously directly from the diet (often found in *Brassicaceae* such as cabbage or broccoli) or produced endogenously (mostly tryptophan-derived metabolites). Disruption of AhR signaling resulted in a loss of ILC3 and a decrease in IL-22 production, most likely due to loss of AhR binding to the *il22* promoter region (11–13). Consequently, AhR-deficient mice or mice fed a AhR-ligand deficient diet failed to clear intestinal bacterial infections (11–13). The importance of endogenous AhR ligands is further emphasized by constitutive intestinal expression of cytochrome P4501 (CYP1) enzymes, which are necessary for detoxification and degradation of AhR ligands (28, 29). Constitutive expression of Cyp1a1 resulted in reduced ILC3 numbers, decreased Th17 differentiation, and increased susceptibility to intestinal infection (30). This was mediated by constant depletion of endogenous AhR ligands and could be counterbalanced by increasing the availability of external dietary ligands. Interestingly, internal ligands may be provided by commensal bacteria such as *Lactobacilli*, which may help to prevent infections with *Candida albicans* through increased expression of IL-22 (31). However, this effect was dependent on unrestricted access to dietary tryptophan. Thus, the dietary availability of both RAR and AhR ligands is crystalizing as a key feature controlling ILC3-mediated barrier protection against bacterial pathogens (Figure 1A).

### Lipid Mediators

Lipids are a third class of dietary components in direct control of immune functions (32). The majority of immunologically active lipid mediators, including PGs, leukotrienes (LTs), and lipoxins (LXs), are derived from arachidonic acid (AA). AA is synthesized from the essential polyunsaturated fatty acids (PUFAs) α-linolenic acid (18:3) and linoleic acid (18:2), critically linking the dietary supply of lipids to the ability to generate lipid signaling molecules. The importance of these lipid mediators in ILC function is multifaceted. PGD_2_ receptor [chemokine receptor homologous molecule expressed on Th2 lymphocytes or (CRTH2)] is used as a defining feature of human ILC2, pointing to a particular importance of PGs in the regulation of ILC2 responses (33). Indeed, PGD_2_ acts as a strong chemoattractant and activator of ILC2 in the context of inflammation in mouse and man, but is negligible for their maintenance (34, 35). These features make CRTH2 a promising target in clinical trials for the treatment of asthma (36). Additional lipid signaling molecules of ILC2-mediated pathology include LT, which, in concert with IL-33, act to amplify ILC2-driven airway inflammation (37, 38). Leukotriene D_4_ (LTD_4_) also mediates barrier protection by promoting ILC2-derived IL-4 and induction of Th2 cell responses in helminth infections (39).

The inflammatory potential of some lipid mediators is counterbalanced by the pro-resolving function of others. LXA_4_ is capable of suppressing PGD_2_ driven IL-13 secretion by ILC2, while PGI_2_ suppresses ILC2 functions in both mice and humans (40, 41). PGI_2_-receptor (IP)-deficient mice display features of uncontrolled ILC2-mediated lung pathology upon intranasal challenge with the fungus *Alternaria alternata* (41). In this study Cicaprost, a PGI_2_ analog, showed promising therapeutic potential as it limits the secretion of pro-inflammatory cytokines from ILC2. A more indirect effect was observed for maresin-1, a pro-resolving mediator derived from Omega-3 PUFAs, which suppresses ILC2-derived IL-5 and IL-13 through the induction of T regulatory cells (Tregs) and TGF-β secretion (42). Finally, by binding to the receptor EP_4_, PGE_2_ can prevent systemic inflammation through increased ILC3-derived IL-22 (43). Consequently, lipid mediators are essential modulators of ILC-mediated barrier immunity in general, but in particular of ILC2-mediated immune functions (Figure 1A).

### ILC in Control of Host Metabolism

ILC2 are a cell type considered to be dedicated to the defense and maintenance of the tissue barrier. However, the first description of ILC2 as a unique cell population was as resident cells of the mesenteric fat (44). This finding eventually led to the discovery that ILC2 are a major component of healthy but not obese adipose tissue, important for the maintenance of a lean state in mice and humans (8–10, 45). Non-obese adipose tissue-infiltrating immune cells mainly consist of alternatively activated macrophages (AAM), eosinophils, Tregs, and ILC2 (8, 45–47). This homeostasis is perturbed in high-fat diet (HFD) induced obesity, where a loss of ILC2 coincides with increases in ILC1, neutrophils, inflammatory macrophages, and activated T cells (8, 45, 46, 48, 49). The importance of ILC2 in regulation of host metabolism was further demonstrated by depletion of ILC2 in wild-type or in obese Rag1^−/−^ mice, which increased weight gain and glucose intolerance (10). Additionally, gain- and loss-of-function studies have verified the importance of IL-33 or IL-25 stimulated ILC2 in white adipose tissue homeostasis. Treating obese mice with IL-33 or IL-25 results in weight loss and increased glucose tolerance, while the opposite effect is observed in mice lacking IL-33, which demonstrates the importance of activated ILC2 for the overall metabolic fitness of the organism (8–10).

Multiple mechanisms of action have been proposed for ILC2 to control host metabolism. One includes that type 2 cytokines, such as IL-5 and IL-13, induce AAM and the accumulation of eosinophils. Both cell types promote the beiging of white adipocytes (9, 50), AAM potentially through the release of noradrenalin and induction of thermogenesis in brown and lipolysis in white adipocytes (51). Lack of IL-13 is associated with weight gain, reduced eosinophils and AAM in adipose tissue (10). However, the direct involvement of AAM in adipocyte metabolism was lately challenged (52), hence more research is needed to identify the exact modes of action. Besides these effects, eosinophil and ILC2-derived IL-4 directly prompted the proliferation and differentiation of adipocyte precursors into beige adipocytes (9). Beige fat is characterized by large quantities of mitochondria and expression of uncoupling protein 1 (UCP1) (53, 54). Here, IL-33 mediated release of methionine-enkephalin from ILC2 increases UCP1 expression, thermogenesis and beiging of white adipose tissue. This results in accelerated energy expenditure preventing obesity and metabolic inflammation (8). However, IL-33 may be able to directly induce beiging of white adipose tissue by regulating the appropriate splicing of *ucp1* mRNA (55). In support of a direct mode of action, IL-33 can prevent adipose tissue inflammation through induction of lipolysis (56).

Although the majority of reports investigated the role of ILC2 in control of host metabolism, one study emphasized the importance of IL-22 expression from ILC3 and T cells for the prevention of diabetes and obesity. Mice displaying genetic ablation of the IL-22 receptor gene were prone to development HFD induced obesity and insulin resistance (57). IL-22 promoted the expression of genes involved in triglyceride lipolysis and fatty acid oxidation (FAO) in adipocytes. Treatment of obese mice with IL-22 suppressed TNF-α expression in visceral adipose tissue (VAT) and improved insulin resistance (57). Finally, a functional switch from cytotoxic to IFN-γ producing ILC1 may promote the inflammatory state in obesity. This may be mediated by simultaneous activation of classically activated macrophages and inhibition of IL-33-induced ILC2 in VAT by IFN-γ (48, 58, 59). Thus, the accumulation of these data suggests a role of ILC beyond the guarding functions at barrier sites, in the maintenance of host metabolism and prevention of obesity-associated inflammation (Figure 1B).

## Metabolic Regulation of ILC

A prerequisite to a better understanding of the regulation of the immune system by dietary components and metabolites is to reveal the metabolic constrains fueling immune cells. Recent advances were made to understand the metabolism of T cells. Naïve T cells are quiescent before activation and rely on FAO and oxidative phosphorylation (OXPHOS) involving the mitochondria (60). Upon antigen encounter and activation, the metabolic requirements of T cells rapidly adapt to match a high energy demand required for proliferation, growth, and cytokine production (61, 62). This is achieved by a switch to aerobic glycolysis, a metabolic process that generates less ATP molecules than OXPHOS but is performed at a faster rate (62). After pathogen clearance, remaining T cells become long-lived memory T cells, which in contrast to effector T cells mainly depend on mitochondrial FAO for persistence and function (62). Despite the relative abundance of data available describing the metabolic control of T cells little data exist revealing the metabolic regulation of ILC.

### Metabolic Regulation of ILC1

**Figure 2:**
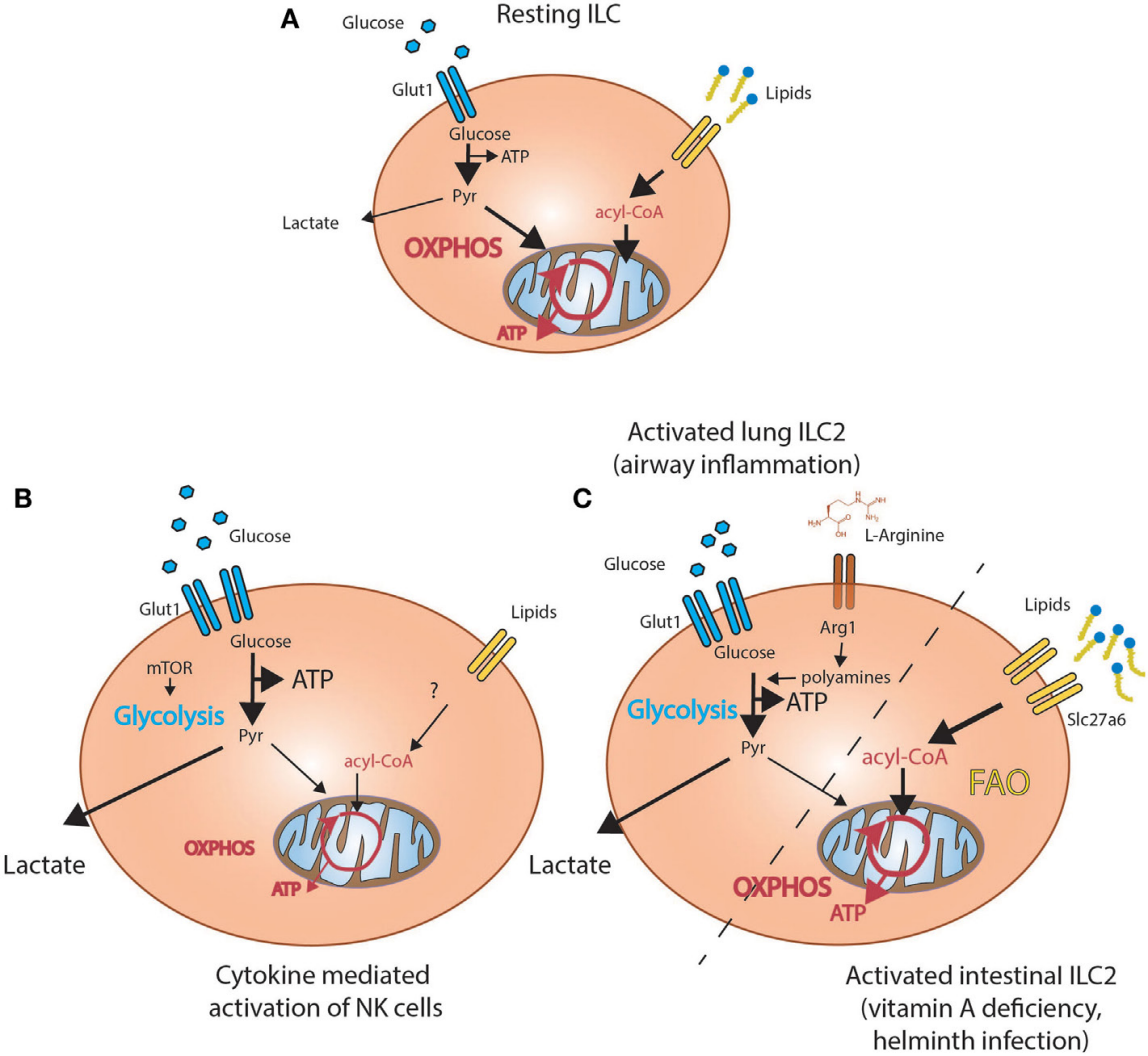
Metabolic programs active in innate lymphoid cells (ILC). **(A)** Resting ILC predominately rely on oxidative phosphorylation (OXPHOS) and take up lipids from the environment. **(B)** Activation of lung ILC2 in the context of airway inflammation (left side) results in increased glycolysis driven by conversion of arginine by Arginase-1 (Arg1) into polyamines. Activation of intestinal ILC2 in the context of helminth infections or vitamin A deficiency (right side) increases uptake of lipids from the environment and results in increased fatty acid oxidation (FAO)-dependent oxidative phosphorylation (OXPHOS). **(C)** Cytokine-mediated activation of NK cells results in increased uptake of glucose and increased mammalian target of rapamycin (mTOR)-dependent glycolysis.

The primary role of ILC1 including NK cells is the protection of the host against intracellular pathogens and tumors. In analogy to naive T cells, freshly isolated splenic NK cells preferentially use OXPHOS over glycolysis prior to activation (63) (Figure 2A). Activation of splenic NK cells *in vivo* with poly(I:C) (activating toll-like receptor 3 and Rig-I) resulted in increased uptake of the glucose analog 2-NBDG and expression of the l-amino acid transporter CD98, suggesting dependence on glutamine and glucose metabolism upon activation. Indeed, metabolic profiling of *in vitro* activated NK cells demonstrated induction of both glycolysis and OXPHOS, although extended stimulation with high dose IL-15 (100 ng/ml for 18–120 h) was required for significant induction of glycolysis (63, 64). These findings apply to human NK cells, suggesting a conserved mode of action in mammals (65). The bioenergetic adaptation of NK cells upon activation is regulated by the mammalian target of rapamycin (mTOR) and both *in vitro* and *in vivo* stimulation with IL-15 or poly:IC, respectively, increased mTOR activity (64, 66). Targeting mTOR with rapamycin decreased expression of both IFN-γ and granzyme B *in vivo* and *in vitro* but also in cultured human NK cells (64, 66). The functions of mTor are cell intrinsic, since acute genetic deletion of mTOR in NKp46 expressing cells and transfer into wild-type mice, revealed a metabolic disadvantage in nutrient uptake and activation (64). Thus, in analogy to T cells, the activation of NK cells favors glycolysis, a switch recently shown to be controlled by the transcription factor Srebp (67) (Figure 2B). Interestingly, blocking mTOR signaling in the resolution phase of inflammation has the opposite effect and promotes the survival of memory NK cells through stimulation of autophagy as does the treatment of mice with the anti-diabetic drug metformin (68, 69).

### Metabolic Regulation of ILC2

Nonetheless, whether induction of glycolysis upon activation is a defining feature of all ILC populations remains unclear. A recent study demonstrated that amino acid metabolism might play a central role for the regulation of ILC2. Arginase-1 (Arg1), essential for the metabolism of l-arginine into urea and ornithine, is expressed by progenitor and mature ILC2. In a model of allergen-induced airway inflammation, genetic deletion of Arg1 in all lymphocytes reduced the amount and proliferation of ILC2 and inflammation (17). The overall reduction of ILC2 was caused by decreased conversion of l-arginine into polyamines, which impaired aerobic glycolysis fueling ILC2 proliferation and accumulation (17). By contrast, deletion of Arg1 in macrophages and neutrophils had no effect on overall pathology. This supports the idea that ILC2-specific expression of Arg1 is a critical driver of airway inflammation and was the first report demonstrating a potential role of glycolysis for the pathogenicity of lung resident ILC2 (Figure 2C).

Yet, this finding appears to contradict other studies suggesting a gene expression pattern enriched for FA metabolism in intestinal ILC2 (70). In support of this assumption, intestinal ILC including ILC2 acquire high amounts of FA from the environment in comparison to other tissue-resident cell types such as Tregs (18). In particular, proliferation and accumulation of ILC2 in the context of malnutrition caused by vitamin A deficiency was fueled through increased uptake of extracellular lipids. Supporting the idea of a preferential dependence on FA metabolism, inhibition of systemic FAO by treatment with etomoxir impaired accumulation of ILC2, the production of IL-5 and IL-13 and ablated anti-helminth immune responses (Figure 2C) (18). Similar effects were observed by inhibition of FA uptake by the lipase inhibitor orlistat but not upon impairment of systemic glycolysis. Thus, proliferation and effector functions of ILC2 may be based on a unique FA-fueled metabolic program functioning in settings of low glucose availability and malnutrition.

## Concluding Remarks: Metabolic Control of ILC as Coordinated Program of Barrier Defense?

Taken together, can we identify a common nominator to understand the specific function of each ILC subset by linking the dietary and metabolic control to the corresponding effector function? One obvious difference is the relative dependence of ILC3 on dietary components, such as AhR ligands or vitamin A metabolites. Thus, the functionality of ILC3 maintaining barrier integrity and fighting intestinal pathogens appears to be critically linked to the availability of food-derived metabolites. Although, few data are available on the metabolic control of ILC3, gene expression analysis identified carbohydrate metabolism and glycolysis as a potentially defining metabolic feature of ILC3 (71). Lack of RA results in downregulation of genes involved in glycolysis (18), which could explain the subsequent loss of ILC3 in vitamin A deficiency. In addition, AhR deficiency may increase FAO and protect against HFD-induced obesity (72). Accordingly, dietary-derived tryptophan and vitamin A metabolites may both fuel glycolysis in ILC3, linking the effector function and the metabolic program of ILC3 to dietary availability. Along the same lines, anti-viral immunity mediated by ILC1 likewise appears to depend on glycolysis. Thus, we propose that acute infection-induced ILC predominately use glycolysis to mediate barrier protection against invading pathogens and that this function is closely coupled to nutritional availability. By contrast, the maintenance and function of damaged-induced ILC2 appears to be controlled by host-derived metabolites, lipids in particular. Tissue damage caused by helminth infections requires ILC2 activation and repair. Assuming that such responses are critically dependent on FA availability, lipid mobilization may be crucial to fuel ILC2 functions, which as a side effect may prevent the development of metabolic syndrome. Furthermore, different metabolic constrains in obesity, such as high blood glucose may impose on the metabolic maintenance of adipose ILC2, which ultimately results in a loss of these cells and aggravation of metabolic inflammation. Finally, a different physiological context of ILC2 activation outside helminth-induced tissue repair could explain a divergent metabolic program of allergen-induced ILC2 (17). In conclusion, the metabolic control of ILC and the corresponding effector functions may be intimately intertwined, which offers a new approach to study ILC responses by unraveling their metabolic profile.

## Author Contributions

All authors listed have made a substantial, direct, and intellectual contribution to the work and approved it for publication.

## Conflict of Interest Statement

The authors declare that the research was conducted in the absence of any commercial or financial relationships that could be construed as a potential conflict of interest.

## Abbreviations

AA, arachidonic acid; AAM, alternatively activated macrophage; AhR, aryl hydrocarbon receptor; AHR, airway hyperreactivity; Arg1, Arginase 1; CAM, classically activated macrophage; CRTH2, chemokine receptor homologous molecule expressed on Th2 lymphocytes; CYP1, cytochrome P4501; DC, dendritic cells; FAO, fatty acid oxidation; IFN-γ, interferon-γ; IL, interleukin; ILC, innate lymphoid cells; HFD, high-fat diet; LTD_4_, leukotriene D4; LTi, lymphoid tissue inducer cell; LTs, leukotriens; LXA_4_, lipoxin A4; LXs, lipoxins; MetEnk, methionine-enkephalin; OXPHOS, oxidative phosphorylation; PGD_2_, prostaglandin D2; PGE_2_, prostaglandin E2; PGs, prostaglandins; PUFA, Omega-3 polyunsaturated fatty acid; RA, retinoic acid; RAR, retinoic acid receptor; RXR, retinoic X receptor; TCDD, 2,3,7,8-tetrachlorodibenzo-p-dioxin; Treg, cell T regulatory cell; UCP1, uncoupling protein 1; VAT, visceral adipose tissue.
